# DNA Damage: A Sensible Mediator of the Differentiation Decision in Hematopoietic Stem Cells and in Leukemia

**DOI:** 10.3390/ijms16036183

**Published:** 2015-03-17

**Authors:** Cary N. Weiss, Keisuke Ito

**Affiliations:** Ruth L. and David S. Gottesman Institute for Stem Cell and Regenerative Medicine Research, Departments of Cell Biology/Stem Cell Institute and Medicine, Albert Einstein Cancer Center and Diabetes Research Center, Albert Einstein College of Medicine, Bronx, NY 10461, USA; E-Mail: cary.weiss@med.einstein.yu.edu

**Keywords:** reactive oxygen species, DNA damage, hematopoietic stem cells, differentiation

## Abstract

In the adult, the source of functionally diverse, mature blood cells are hematopoietic stem cells, a rare population of quiescent cells that reside in the bone marrow niche. Like stem cells in other tissues, hematopoietic stem cells are defined by their ability to self-renew, in order to maintain the stem cell population for the lifetime of the organism, and to differentiate, in order to give rise to the multiple lineages of the hematopoietic system. In recent years, increasing evidence has suggested a role for the accumulation of reactive oxygen species and DNA damage in the decision for hematopoietic stem cells to exit quiescence and to differentiate. In this review, we will examine recent work supporting the idea that detection of cell stressors, such as oxidative and genetic damage, is an important mediator of cell fate decisions in hematopoietic stem cells. We will explore the benefits of such a system in avoiding the development and progression of malignancies, and in avoiding tissue exhaustion and failure. Additionally, we will discuss new work that examines the accumulation of DNA damage and replication stress in aging hematopoietic stem cells and causes us to rethink ideas of genoprotection in the bone marrow niche.

## 1. Introduction

Hematopoietic stem cells (HSCs), like other stem cell populations, are defined by their ability to self-renew and to differentiate [1,2]. A phenotypically identified and isolated HSC is capable of contributing to all of the mature hematologic lineages and is capable of replenishing the HSC pool after lethal irradiation [3,4,5]. Additionally, the differentiation hierarchy that progresses from the HSC, to the progenitor, to a functionally mature effector cell is well understood. These features make HSCs the best-characterized adult stem cell population. Thus, the hematopoietic system, including HSCs and hematological malignancies, serves as an essential investigative avenue in stem cell biology [6].

Among cellular components, the genome is particularly susceptible to damage, which can result from spontaneous reactions in the nucleus, oxidative damage due to metabolic byproducts or from extrinsic agents, or replication associated defects [7,8]. Because the genome must be maintained for the life of the cell, and because it can be copied and propagated into daughter cells, damage to DNA brings more severe consequences than damage to replaceable cellular macromolecules. Of course, accumulated DNA damage is essential in the development of malignancies, and DNA damage accumulated in HSCs and progenitors is responsible, in part, for hematological malignancies [9,10]. Stem cells, including HSCs, may be particularly susceptible to DNA damage due to their longevity [11].

HSCs replenish themselves and provide mature blood cells, and so, HSCs are thought to be responsible for safeguarding the genomic integrity of the hematopoietic system [12]. An unrepaired genetic lesion maintained in an HSC is capable of being spread throughout the HSC pool, through self-renewing divisions, as well as propagating to all of the hematologic compartments, through differentiation [11]. It is thought that HSCs remain quiescent and reside in the hypoxic bone marrow niche in order to avoid reactive oxygen species (ROS) and other stresses to the genome [13,14,15,16,17]. One might hypothesize that HSCs also exhibit uniquely robust DNA damage repair mechanisms, in order to further fortify their genome. However, much evidence suggests an opposing hypothesis—the consequences of erroneous DNA damage repair in an HSC could be so severe that it is preferable for an HSC faced with oxidative or other genotoxic stress to differentiate, removing it from the stem cell pool and preventing the dissemination of deleterious mutations. The pathways that underlie this decision have been coined the “ROS rheostat [18,19,20,21,22,23,24,25,26]”.

We have previously reviewed the effects of cellular metabolism and oxidative stress, and of the DNA damage response on HSC maintenance [20,26]. In this review, we will investigate three recent papers that have led us to reexamine the contributions of DNA damage repair to the development of malignancy, and also to reconsider the notion that HSCs are privileged to avoid genotoxic stress and the accumulation of DNA damage [27,28,29]. In the first, Santos, *et al.*, the authors explored a role for the DNA damage response and DNA damage resistance in promoting the development of hematological malignancy [27]. This work is the culmination of accumulated evidence that has suggested a mechanism through which HSCs differentiate when faced with genotoxic stressors, likely to avoid the propagation of mutations in a population capable of self-renewal [18,19,20,21,22,23,24,25,26]. The second publication, Beerman, *et al.*, describes the accumulation of DNA damage with age in quiescent HSCs due to an inefficient response to DNA damage [28]. The accumulation of damage and inefficient damage repair seems to be unique to quiescent HSCs in the hematopoietic system, is abrogated upon cell cycling, and may contribute to declining HSC function with age. Finally, in Flach, *et al.*, the authors have identified accumulated DNA damage markers associated with replication stress in old HSCs that is distinct from DNA strand breaks [29]. In this publication, the Passegué group identifies downregulation of *Minichromosome maintenance* (*Mcm*) family genes as a mediator of replication stress in old HSCs, which contributes to the reduced function of aged HSCs. Together, these publications contribute greatly to our understanding of the role of DNA damage in aging and malignancy, and identify a number of pathways worthy of further investigation for their potential clinical importance.

## 2. The DNA Damage Response as a Potent Oncogenic Driver

**Figure 1:**
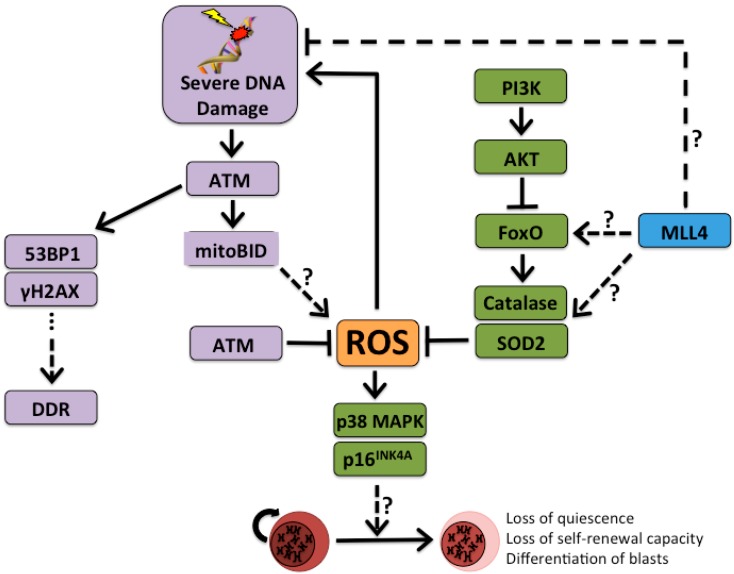
The ROS rheostat of hematopoietic stem cell (HSC) maintenance. Accumulation of DNA damage and genotoxic oxidative stress contributes to a common pathway that leads to loss of self-renewal capacity of HSCs and leads HSCs to exit their quiescent state. This contributes to the gradual decline of functional HSCs in the bone marrow. Mixed lineage leukemia 4 (MLL4) activates forkhead box O (FoxO) targets through an unknown mechanism, and MLL4 expression is shown to be protective in the MLL1-AF9 (ALL1-fused gene from chromosome 9, or MLLT3) of AML by reducing the accumulation of ROS and, thus, DNA damage. *Mll4*-deficiency may also contribute to DNA damage through a ROS-independent mechanism. DNA damage results in the activation of ATM and, subsequently, DDR. Accumulation of γH2AX and co-localization with 53BP1 serve as markers of DDR, as in Flach, *et al.* Under normal conditions, ATM helps to maintain ROS at low levels. However, in the face of severe DNA damage ATM contributes to the accumulation of ROS and loss of quiescence in HSCs. ATM, ataxia telangiectasia mutated; FoxO, forkhead box O; DDR, DNA damage response; γH2AX, phosphorylated histone H2AX; MLL4, mixed-lineage leukemia 4; mitoBID, mitochondrial BH3 interacting-domain death agonist; MLL4, mixed-lineage leukemia 4; p38 MAPK, p38 mitogen-activated protein kinases; PI3K, phosphoinositide 3-kinase; ROS, reactive oxygen species; SOD2, superoxide dismutase 2; TP53BP1, tumor suppressor p53-binding protein 1. p16INK4A, cyclin dependent kinase inhibitor 2A; AKT, protein kinase 3. Solid arrows represent known mechanisms; dashed arrows labeled with question marks represent unknown mechanisms.

Recently, the Nussenzweig group presented strong evidence in support of the hypothesis that DNA damage response (DDR) pathways might be cancer protecting in some developing malignancies [27]. This work presents an interesting contrast to other studies that demonstrate a cancer-protective role for DDR-associated genes, such as those associated with the ATM-CHK2-p53 (Ataxia telangiectasia mutated, Checkpoint kinase 2, and p53, respectively) pathway, and complicates our understanding of the role of DNA damage in malignancy [30,31]. In their paper, Santos, *et al.* identified mixed lineage leukemia 4 (MLL4) as a positive regulator of genes responsible for safeguarding cells against damaging ROS, and observed increased differentiation in *Mll4*^−/−^ HSCs, consistent with previously reported observations of the effects of accumulated oxidative damage on HSCs [19,23,25,32]. Retroviral expression of MLL1-AF9 (ALL1-fused gene from chromosome 9, or MLLT3) in several hematopoietic compartments results in the generation of acute myeloid leukemia (AML) upon transplantation in mice, and has been used as a model to study AML and leukemia stem cells (LSCs, also known as leukemia-initiating cells) [33,34,35,36,37,38]. The Nussenzweig group demonstrated that *Mll4*-deficient colonies that express the MLL1-AF9 fusion oncogene contain mature cells, rather than undifferentiated blasts, and are unable to generate AML in mice upon transplantation. Further, they demonstrated that the *Mll4-*deficient cells expressing MLL1-AF9 displayed higher levels of ROS, as measured by CellROX staining, and higher levels of DNA damage, as indicated by increased Kap-1 staining and γH2AX foci. Treating *Mll4-*deficient MLL1-AF9 transformed cells with antioxidants (*N*-acetyl-l-cysteine, or NAC) partially rescues the leukemic phenotype. Additionally, ectopic expression of forkhead box O3 (FoxO3) rescues the leukemic phenotype, suggesting that differentiation in transformed MLL4-deficient cells occurs through target genes for FoxO family of transcription factors, which are responsible for mediating oxidative stress and have been studied in the context of maintaining HSC quiescence (Figure 1) [23,25,32]. Similarly, the Nussenzweig group demonstrated that oxidative stress contributes to differentiation of MLL1-AF9 transformed cells in the context of hydrogen peroxide treatment. Further, DNA damage alone, outside of the context of oxidative stress, is cytoprotective against the effects of the MLL1-AF9 fusion oncogene. Using a *Brca1*-deficient mouse model, which exhibits DNA damage independent of oxidative stress, and, separately, in a model of DNA double strand breaks using an ectopically expressed restriction endonuclease, Santos, *et al.* demonstrated that DNA damage alone can also lead to differentiation and exhaustion of MLL1-AF9 transformed leukemia. When DNA damage persists and is detected by cell-cycle checkpoint machinery, leukemic cells enter a differentiation program and lose some of their malignant potential. In their model of MLL1-AF9 transformation, differentiation that results from accumulated DNA damage is dependent on the cell cycle checkpoint protein *p21^Cip^* (Cdkn1a) [27]. When *p21* is lost in the context of MLL1-AF9, cells are resistant to DNA damage associated growth inhibition and differentiation, consistent with previous reports that cell cycle elongation contributes to differentiation [39,40].

The demonstration that pathways that work to maintain genomic integrity are protective in this model of AML presents some interesting prospects for the treatment of these malignancies, namely through inhibition of the DNA damage repair initiators ataxia telangiectasia mutated (ATM) and ataxia telangiectasia and Rad3-related (ATR). Treatment with these inhibitors contributes to an accumulation of mature cells and a loss of blasts in the context of MLL1-AF9 transformed cells, and MLL1-AF9 transformed *Atm*^−/−^ bone marrow is incapable of maintaining leukemic self-renewal without differentiation [27]. In fact, an earlier study utilizing a model of hypomorphic ATR and ATR inhibitors supports the Nussenzweig group’s findings [41]. Additionally, Santos, *et al.* represents an advance in our understanding of the roles of ROS, DNA damage sensing, and cell-cycle checkpoints in differentiation and cell fate decisions in leukemia and in HSCs. There is much evidence supporting the idea that HSCs, when faced with DNA damage or genotoxic stress, differentiate to lineage-committed progenitors, and this may serve as a method to escape propagating damaged genetic information throughout the HSC pool and the hematopoietic system. Described another way, hematologic malignancies thrive on the failure of this escape mechanism, choosing DNA repair over differentiation, in order to maintain their self-renewal.

## 3. Sensing Stress and Quitting Quiescence

As previously mentioned, HSCs are particularly susceptible to DNA damage because of their longevity. Additionally, DNA damage in HSCs can be propagated throughout the HSC pool or to mature effector cells through self-renewing and differentiation divisions, respectively. In the face of genotoxic stress the accumulation of ROS serves as a rheostat in the differentiation decision, integrating information from a number of pathways (Figure 1).

Intracellular ROS are byproducts of aerobic metabolism in mitochondria, and may also originate from other organelles [42,43]. DNA is highly susceptible to oxidative damage, which can result in single and double strand breaks (SSBs and DSBs), base and sugar-moiety oxidation, strand crosslinks and the generation of abasic sites [7,8,17,20,44,45]. The initial steps in detection of strand breaks do not require discussion here. Phosphatidylinositol 3 kinase-related kinase (PIKK) family members, the checkpoint kinases ATM and ATR, are recruited to the site of the damage and activated. These enzymes phosphorylate a number of targets initiating signaling cascades that mediate cell cycle arrest and DDR [46,47]. ATM can also be activated to induce DDR in the context of oxidative stress, thus serving as sensor of reactive oxygen species. ATM itself can be oxidized, yielding a disulfide-crosslinked ATM dimer that activates DDR in the absence of DSBs, in contrast to the active monomer generated in the context of DSBs [48]. ATR activation by SSBs, which result from oxidative stress-induced abasic sites, utilizes a unique Apurinic/apyrimidinic (AP) endonuclease 2 (APE2)-dependent mechanism [49]. APE2 participates in the removal of the damaged 3' terminus, generating a 3'–5' SSB end resection and allowing for the recruitment of ATR. Among the downstream targets of ATM are Histone H2AX (H2A histone family, member X), which is phosphorylated at serine 139 to become phosphorylated histone H2AX (γH2AX), and tumor suppressor p53 binding protein 1 (TP53BP1). Due to these interactions, γH2AX staining is commonly used as an indirect marker of DNA damage, and co-localization of TP53BP1 can sometimes be detected in γH2AX foci [27,29]. Studies have demonstrated that ATM is also a powerful mediator of ROS homeostasis, which contributes to its ability to mediate the differentiation decision in response to genotoxic stress. *Atm*^−/−^ mice suffer from an accumulation of ROS that is mediated by treatment with the antioxidant NAC [50,51,52,53]. The mechanism through which ATM suppresses the accumulation of ROS is unclear. Under normal conditions, phosphorylation of BH3-interacting domain death agonist (BID) by ATM restricts BID to the nucleus and is essential in maintaining HSC quiescence, possibly through and ROS dependent mechanism [21,22]. In the context of severe DNA damage, BID and phosphorylated BID translocate to the mitochondria and result in the accumulation of ROS. Additionally, *Atm*^−/−^ mice have a reduced reconstitution capacity of HSCs, and elevated levels of the Rb and p53 activators p16^INK4A^ (cyclin-dependent kinase inhibitor 2A) and p19^ARF^ (alternate reading frame tumor suppressor), which contribute to the loss of HSCs—both observations can be rescued with NAC treatment, demonstrating a role for ROS deregulation in the development of this phenotype [18,54] In the context of elevated ROS, p16^INK4A^ levels seem to regulated by the intermediary p38 mitogen-activated protein kinases (p38 MAPK), and p38 MAPK inhibition rescues the hematopoietic phenotype observed in *Atm*^−/−^ mice [19].

The family of forkhead box binding O (FoxO) proteins is one of the earliest families of transcription factors that have been implicated in sensing and responding to oxidative stress in HSCs. In 2007, both the Gilliland group, using a triple conditional knockout of *FoxO1*, *FoxO3* and *FoxO4*, and the Hirao and Suda groups, using *FoxO3a*-deficient mice, reported on the necessity of FoxOs in the maintenance of HSCs [23,25]. The FoxO transcription factors are responsible for positively regulating the expression of oxidative stress mitigating proteins, including superoxide dismutase and catalase enzymes [23,24,25]. *FoxO*-deficient mice exhibit a decrease in HSC number, a loss of HSC quiescence, and HSCs from *FoxO*-deficient mice are incapable of reconstituting bone marrow upon transplantation [25]. These defects can be rescued through treatment with NAC and are the result of reduced expression of ROS neutralizing enzymes [25]. Similarly, *FoxO3a*^−/−^ mice show reduced levels of the ROS scavenging enzymes Superoxide dismutase 2 (SOD2) and catalase, which are positively regulated by FoxO3a, and show loss of HSC function [23,32]. FoxO family proteins are inhibited by exclusion from the nucleus as a result of phosphorylation by AKT, downstream of the PI3K-AKT phosphorylation cascade [55]. The negative effects to HSC number and function that are observed when FoxOs are inhibited strongly suggest a role for accumulated ROS in the decision for an HSC to differentiate.

## 4. Replication: A Source for Rejuvenation or Stress in Aged Hematopoietic Stem Cells (HSCs)

**Figure 2:**
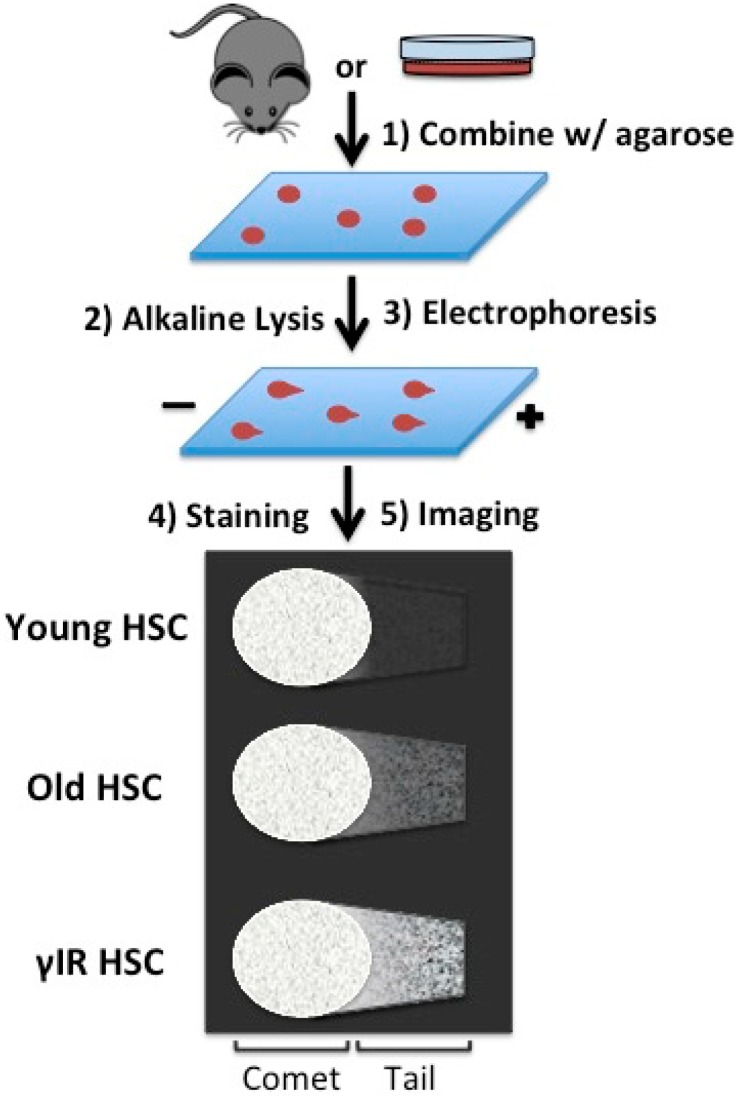
The alkaline comet assay. The alkaline comet assay allows for the direct microscopic measurement of DNA damage. Isolated cells are mixed with low-temperature gelling agarose and applied to a glass slide. Cells are lysed under alkaline conditions in order to detect single and double strand breaks, though a number of lysis procedures have been described for other purposes. After a brief electrophoresis and staining, DNA damage can be visualized microscopically. High molecular weight DNA, reflecting undamaged DNA, remains in the comet, whereas damaged DNA is susceptible to the electrophoretic field and is found in the tail. A number of methods for quantifying and describing the tail to comet relationship have been described. In Beerman, *et al.* the authors describe a higher frequency of HSCs with moderate-to-severe DNA damage in aged mice as compared to younger mice [28]. Damage is also more severe among aged hematopoietic stem cells as compared to aged hematopoietic progenitors.

In the past year, the Rossi and Passegué groups both published dogma-challenging work on the genoprotection of aging HSCs in the bone marrow niche [28,29]. In mice, HSC aging contributes to reduced engraftment upon transplantation, and impaired lymphoid differentiation upon transplantation and in steady state hematopoiesis [56]. In humans, HSC aging is thought to predispose HSCs to the accumulation of malignancy driving mutations. Through studies examining γH2AX, a marker of DNA damage sensing, and examining genetic models of DDR deficiency, it has already been suggested that DNA damage accumulates in old HSCs and contributes to their reduced function with age [57,58,59]. In their recent work, the Rossi group took advantage of the single-cell alkaline comet assay, an older technique which provides a direct measure of DNA single and double strand breaks (Figure 2), in order to demonstrate increased levels of accumulated damage in aged HSCs [60,61,62]. In this assay, isolated cells are mixed with low-temperature gelling agarose, deposited on a glass slide, lysed under alkaline conditions, and subjected to a brief electrophoresis [60]. Staining the slides with a DNA labeling compound, such as SYBR Green, allows for DNA to be visualized microscopically and analyzed with specifically developed software—the tail of the comet in this assay consists of leading strands of DNA that result from single and double strand breaks, whereas unbroken DNA fails to migrate due to its high molecular weight [60]. In Beerman, *et al.*, the authors demonstrate that there are more cells with accumulated moderate-to-severe DNA damage in HSCs from aged mice (24–26 months) as compared to HSCs from young mice (3–4 months), though moderate to severe damage can be detected in young HSCs and undamaged HSCs can be identified in the aged cohort [28]. While hematopoietic progenitors from aged mice also show evidence of accumulated DNA damage, the damage is clearly more severe in the HSC compartment, as evidence by a higher percentage of moderate-to-severe damaged HSCs, suggesting that HSCs are especially susceptible to accumulating DNA damage [28]. Accumulated DNA damage in old HSCs is repaired when the HSCs are caused to cycle. Old HSCs that are cultured in cytokine rich medium for 24 h resemble similarly treated young HSCs, and even display less DNA damage compared to young HSCs at steady state, suggesting that cell cycle entry represents a potent driver of DNA damage repair, which was absent or reduced in quiescent HSCs [28]. Of note, however, is that aged HSCs display a reduced proliferative potential in long-term culture, presumably after DNA damage has been repaired, as compared to young HSCs. HSCs in aged mice treated with 5-fluorouracil, as well as aged HSCs allowed to engraft upon bone marrow transplantation, display significantly reduced DNA damage [28]. Here too, despite damage repair, aged HSCs have reduced reconstitution capacity with a myeloid bias upon transplantation, similar to those observations made in *in vitro* experiments and consistent with a reduction of HSC function with age. Thus, the DNA damage repair observed upon cell-cycle reentry is not sufficient to completely restore hematopoietic stem cell function.

The Rossi group demonstrated that hematopoietic progenitors, as compared to stem cells, exhibit increased expression of DDR associated genes, which is supported by previous evidence that DDR is tightly linked with cell cycle entry and that progenitors are actively cycling as compared to the largely quiescent HSCs [13,14,63,64]. Fetal liver HSCs, which are actively cycling, as well as young and aged HSCs stimulated to cycle in cytokine-rich medium, demonstrated increased expression of DDR associated genes [28,65]. Interestingly, genes associated with non-homologous end joining (NHEJ), an error-prone mechanism of DNA damage repair, are expressed at similar levels between HSCs and progenitors [28,66]. The authors suggest that NHEJ must not be active in quiescent HSCs, as active NHEJ would be inconsistent with the observed level of DSBs. Instead, they propose a hypothesis that quiescent HSCs are primed to undergo NHEJ upon cell cycle reentry. The authors conclude that DDR pathways are reduced in quiescent HSCs, contributing to the increased DNA damage in young and aged HSCs, as compared to progenitors, observed by the comet assay. Because HSCs remain quiescent for most of adult life they accumulate mutations that limit HSC function with age, despite the repair mechanisms that are initiated upon cell cycle reentry [67].

The persistence of DNA damage and inability to completely restore hematopoietic function, despite activation of DNA repair mechanisms at cell-cycle reentry may be reflected in reports of the accumulation of leukemogenic mutations in HSCs that contribute to the development of “pre-leukemic HSCs” observed by the Majeti group [68,69]. Jan, *et al.* used a previously described strategy to prospectively isolate residual HSCs from AML samples using T-cell immunoglobulin mucin 3 (TIM3) and other surface markers [70,71]. Exome sequencing and transcriptome analysis of AML cells and residual HSCs revealed genetic mutations that are frequently associated with AML in residual HSCs, strongly supporting the existence of pre-leukemic HSCs in the phenotypically normal HSC population [68]. Further, sequencing of colonies formed from single residual HSCs allowed the Majeti group to observe the sequential accumulation of frequently occurring AML mutations in residual HSCs. This work provides strong evidence in support of a model of sequential accumulation of leukemogenic mutations in self-renewing HSCs contributing to the generation of pre-leukemic HSCs and eventually frank leukemia, however no experimental evidence showing that the accumulation of additional mutations in residual HSCs/pre-leukemic HSCs leads to leukemia is provided in this study. While the recent work from the Rossi group does not provide a clinical avenue to avoid or reduce the accumulation of DNA damage in aging HSCs, it does support the hypothesis that leukemogenic mutations accumulate in HSCs in a stepwise fashion. Treatments that target early mutations found in pre-leukemic HSCs would likely result in increased disease free survival in AML and in other hematologic malignancies.

A publication from the Passegué group, also from the past year, suggests that aged HSCs are more susceptible to DNA damage associated with replication stress [29]. This finding draws in to question the strong reparative effects of cell cycling observed in Beerman, *et al.*, and deserves a closer examination.

In Flach, *et al.*, the authors explore the ability of HSCs from aged mice (22–30 months old, identified here as “aged/old HSCs”) to undergo DNA damage repair, as compared to HSCs from young mice (6–12 weeks old, identified here as “young HSCs”) [29]. The authors confirmed that γH2AX is accumulated in aged HSCs, but were unable to observe a significantly elevated tail moment upon alkaline comet assay, as was observed by the Rossi group [27,29]. However, the authors observed that cultured old and young HSCs were similarly capable to clear γH2AX foci after irradiation, albeit old HSCs exhibit an ~8 h delay, and express homologous repair (HR) and NHEJ genes at similar levels to young HSCs [29]. If old HSCs display a similar ability to cope with DNA damage as young HSCs, this raises the question of what is responsible for the accumulation of γH2AX observed by many groups in aged HSCs? Further investigation would clarify this point.

ATR and γH2AX foci have also been reported to accumulate at replication forks, in a DNA damage independent mechanism (Table 1) [45,72]. Consistently, the Passegué group observed signs of increased single-stranded DNA in aged HSCs, namely increased staining for the single-stranded DNA binding proteins ATR interacting protein (ATRIP) and replication protein A (RPA). These findings are suggestive of replication stress in aged HSCs that would explain the increased γH2AX foci, and explain the delayed cell division kinetics observed in EdU/BrdU (5-Ethynyl-2'-Deoxyuridine and 5-Bromo-2'-Deoxyuridine, respectively) incorporation experiments due to delayed onset of S phase and elongated S phase [29,73]. Aged HSCs were also observed to have delayed first and second divisions in culture. Additionally, the Passegué group reported the accumulation of chromosomal gaps and breaks in the daughter cells of cultured old HSCs. Interestingly, they found no evidence of NHEJ, specifically no chromosomal deletions or translocations, in expanded cultures of old HSCs, in contrast of what one might expect of DDR in HSCs, which are typically quiescent [66,74].

**Table 1:** DNA damage and DDR associated molecules. Flach, *et al.* utilized a variety of antigens to examine DNA damage, DDR, replication stress and ribosome biosynthesis by immunofluorescence [29]. A number of these markers are reproduced and described here.

Antigen		Abbreviation	Indication	Notes	References
Gamma-H2AX		γH2AX	DSB single stranded DNA	Target of ATM/ATR may serve as repressive epigenetic mark in quiescent aged HSCs	[29,75,76,77,78,79]
Tumor suppressor p53-binding protein 1		TP53BP1	DSB	Target of ATM/ATR	[80,81,82]
Phosphorylated checkpoint kinase 1		pCHK1	DNA damage repair	Target of ATR mediates cell cycle arrest	[83,84,85,86,87]
Phosphorylated ataxia-telangiectasia mutated		pATM	DNA damage DSBs	Mediates DDR mediates redox homeostasis in HSCs	[18,22,30,88,89,90,91]
Poly (ADP-ribose)		PAR	Single stranded DNA break	Signals for single strand break repair synthesized by PARP	[92,93]
Replication protein A	RPA		Binds single stranded DNA	Prevents formation of secondary structures during replication	[94,95]
ATR interacting protein	ATRIP		Binds RPA coated single stranded DNA	Associates with ATR, leading to its accumulation at intranuclear DNA damage foci	[96,97,98]
Fibrillarin	FBL		Ribosome biosynthesis	Fibrillarin component of SnRNPs	[99,100,101]
Upstream binding factor	UBF		Ribosome biosynthesis	Upstream binding factor transcription factor of rRNAs	[102]
Nucleolin	NCL		Ribosome biosynthesis	Nucleolin invlolved in ribosome synthesis	[103,104]
Nuclear serine/threonine protein phosphatase 4 catalytic subunit	nPP4c			γH2AX phosphatase	[105,106]

The minichromosome maintenance (MCM) family of genes encodes the subunits of the hexameric MCM DNA helicase, a member of the CDC45–MCM–GINS (cell division control protein 45, minichromosome maintenance, Go-Ichi-Ni-San, respectively) pre-replication complex that is responsible for unwinding DNA at replication forks during the replication initiation [107]. Examination of differentially expressed genes in old HSCs identified the *Mcm2–7* as down-regulated, whereas no other members of the pre-replication complex were deregulated [29]. Treating old HSCs with aphidicolin, a DNA polymerase inhibitor, resulted in dramatic accumulation of γH2AX foci and impaired stem cell function as compared to young HSCs treated with aphidicolin, consistent with observations of replication stress in HeLa cells subjected to MCM knockdown by siRNA [108]. Additionally, knockdown of MCM components in young HSCs replicates the stem cell defects observed in old HSCs in culture and upon transplantation [29]. In fact, treatment of young HSCs with aphidicolin, but without modifying MCM levels, also reduces the reconstitution capacity, suggesting a powerful role for replication stress in HSC function.

In quiescent aged HSCs, in which γH2AX foci cannot be explained by replication stress, the Passegué group identified γH2AX accumulation at nucleoli, the sites of ribosome biogenesis, by fluorescent *in situ* hybridization (FISH) for rDNA genes. The authors suggest that γH2AX scars likely originate from replication stress at rRNA encoding regions, which are difficult to replicate, and may persist because of localization γH2AX phosphatase PP4c in the cytoplasm of quiescent old HSCs [109]. When old quiescent HSCs reenter the cell cycle, nucleolar γH2AX are lost. However, many months after transplantation, when HSCs may have returned to quiescence, nucleolar γH2AX foci can be observed. Interestingly, though no gene regulatory role for γH2AX has been previously identified, γH2AX accumulation at rDNA was associated with decreased expression of ribosomal components, which the authors propose is a non-canonical function of γH2AX to repress transcription in regions undergoing active DNA repair. Decreased ribosomal synthesis in quiescent older HSCs may contribute to decreasing HSC function with age.

This study suggests that identifying a therapeutic strategy to restore MCM expression and activity may prove useful in slowing stem cell decline and rejuvenating an aged HSC compartment. Further, reduction of γH2AX in quiescent aged HSCs might contribute to improved HSC function with age, however additional studies of the role of ribosomal biogenesis stress on quiescent HSCs are needed.

## 5. Conclusions

DNA damage to HSCs that persists in the stem cell compartment can be deleterious to hematopoietic function and can promote malignancy. Because HSCs have the ability to self-renew and to differentiate, they are thought to be responsible for maintaining genomic integrity of the hematological system, and it had been proposed that HSCs were protected from genomic damage by their hypoxic microenvironment and possibly by cell intrinsic factors. Mounting evidence, including the recent works discussed here, suggests that HSCs, due to their longevity and quiescence, are highly susceptible to accumulating DNA damage. Rather than allow damage to persist in the stem cell compartment where damage can be propagated throughout the hematopoietic system, HSCs faced with DNA damage and genotoxic stress, such as ROS, differentiate. This would serve to restrict the ability of damage to move through the hematopoietic population. To that end, DNA damage repair, commonly the error prone NHEJ in HSCs, might be deleterious to the hematopoietic system and can contribute to the development of malignancy. This is supported by Santos, *et al.*, which identifies mechanisms that reduce oxidative stress and repair DNA damage as protective in the MLL1-AF9 model of AML. When DNA repair mechanisms are inhibited, MLL1-AF9 transformed leukemia is pushed towards differentiation and blasts are lost. With supporting clinical evidence that indicates that malignancy is more sensitive to loss of DDR mechanisms than non-malignant tissues, inhibition of DDR may prove to be a valuable therapeutic option in the treatment of AML that could work similarly to all-trans retinoic acid (ATRA) in the treatment of acute promyelocytic leukemia (APL or AML M3), which pushes leukemic cells towards differentiation.

The Rossi and Passegué groups used different measures of genomic integrity to investigate the role of DNA damage in aging. In Beerman, *et al.*, the authors measured DNA damage directly using the alkaline comet assay and concluded that DNA damage accumulates in quiescent aged HSCs and is repaired upon cell cycle reentry. Upon cell cycle reentry, DNA damage profiles by alkaline comet assay in aged HSCs resemble those of young HSCs, however stem cell function is somewhat decreased in aged HSCs. The persistence of DNA damage in HSCs, perhaps a result of the propensity for HSCs to utilize error-prone NHEJ, may contribute to the stepwise accumulation of leukemogenic mutations and the establishment of pre-leukemic HSCs. In Flach, *et al.*, accumulated γH2AX foci in aged HSCs are attributed to replication stress that results from downregulation of replication helicase machinery in aged HSCs. Interestingly, these two publications include some different findings, which will surely be rectified with continued investigation. Further, the Passegué group identified γH2AX scars at rDNA in quiescent aged HSCs. In this context, γH2AX appears to play a previously undescribed epigenetic role by repressing ribosome synthesis. Further investigations may reveal that this ribosome biogenesis stress may contribute to declining HSC function with aging.

Despite their importance in maintaining the genomic integrity of the hematopoietic system, HSCs are uniquely sensitive to DNA damage and the accumulation of mutation. This is due to their longevity and due to decreased DNA damage repair associated with quiescence. Stepwise accumulation of mutations, as well as replication stress is associated with the functional decline of HSCs with age. Accumulated mutations in HSCs sometimes persist, despite mechanisms that work to remove genotoxically stressed HSCs from the stem cell pool through loss of quiescence and self-renewal. For this reason, DDR mechanisms appear to be oncogenic in HSCs. These publications provide new insights into the accumulation of DNA damage in HSCs and the role of DNA damage and replication stress in the functional decline of hematopoiesis, but also suggest additional questions and areas that require further examination.
